# Tumor Immune Microenvironment and Immunotherapy in Non-Small Cell Lung Cancer: Update and New Challenges

**DOI:** 10.14336/AD.2022.0407

**Published:** 2022-12-01

**Authors:** Shuqin Xing, Kaiwen Hu, Yafei Wang

**Affiliations:** ^1^Department of Oncology, Dongfang Hospital, Beijing University of Chinese Medicine, Beijing, China.; ^2^Department of Orthopedics, Dongfang Hospital, Beijing University of Chinese Medicine, Beijing, China.

**Keywords:** non-small-cell lung cancer, tumor immune microenvironment, immunoediting, cancer treatment

## Abstract

Non-small cell lung cancer (NSCLC) is a serious threat to the health of older adults. Despite the significant progress in immunotherapy, effective treatments for NSCLC remain limited. The development of tumors indicates failure in immune surveillance and the successful immune escape of tumor cells. Research on the tumor immune microenvironment (TIME) revealed these opposing immune processes and contributed to the discovery of new methods to suppress the immune escape and restore the immune surveillance functions. This paper aimed to provide updates on the current findings regarding the relevance of TIME in NSCLC treatment. It also aimed to introduce the TIME, immune editing, cancer immunotherapy, and new challenges. Based on the clinical data, the combination of neoadjuvant chemotherapy and immune checkpoint inhibitor (ICI) therapy is suitable for patients with NSCLC who are not eligible to undergo surgery. Combined ICI therapy after epidermal growth factor receptor (EGFR)/tyrosine kinase inhibitor (TKI) therapy should be considered in patients with EGFR mutations. Chemoradiotherapy may increase the density of CD8^+^ lymphocytes, which is significantly associated with better prognosis. For older patients and those with advanced-stage disease, regional tumor treatments, such as stereotactic radiation therapy and percutaneous cryoablation, may be more suitable, but further studies are needed to confirm this. In conclusion, restoring immune surveillance is as important as removing cancerous tissues; further studies that include the use of combined treatment methods, individualized treatment plans, and immunonutrition are warranted.

According to the World Health Organization, cancer is the second leading cause of death worldwide. Although lung cancer is the leading cause of cancer-related death [1], the older adult population has the highest incidence of lung cancer among all the age groups. Less than 0.5% of deaths caused by lung cancer occur in individuals aged <40 years [2, 3]. Non-small cell lung cancer (NSCLC) accounts for approximately 85% of lung cancer cases and includes all types of epithelial lung cancer except small cell lung cancer (SCLC). The main subtypes of NSCLC are squamous cell carcinoma, adenocarcinoma, and large-cell carcinoma. The standard treatments for early NSCLC are surgery, endobronchial therapy, radiation therapy, and adjuvant and neoadjuvant chemotherapies. For advanced NSCLC, in addition to surgery and radiotherapy, combined chemotherapy, molecular targeted therapy, and immunotherapy regimens have been used. In particular, significant progress in the development of molecular targeted therapy and immunotherapy has been achieved in recent years [4, 5]. Although the relative survival rate of patients with NSCLC has been increasing in the past few decades [6], the treatment effect remains poor in most patients with NSCLC, except for those with localized cancer, who can achieve cure [5]. In 2009-2015, the 5-year overall survival rate of patients with NSCLC in the United States was only 24% [7].

The occurrence and development of tumors depend not only on genetic changes in the tumor cells themselves, but also on the surrounding environment, namely, the tumor microenvironment (TME). In particular, the immune cell composition of the TIME plays a vital role. Therefore, in addition to directly eliminating the tumor cells (surgery, radiation, and chemotherapy) and inhibiting their proliferation (chemotherapy and targeted therapy), inducing the immune system to combat cancer (immunotherapy) has become another promising treatment approach [8]. In this article, the concepts of TIME and immunoediting and the current progress in cancer immunotherapy were discussed. Then, the research methods used to explore the TIME of NSCLC and TIME classification were briefly introduced. Next, the relationship between the current treatment approaches for NSCLC used in clinical practice and TIME was provided. Finally, the results of the aforementioned clinical trials were summarized, and our outlook for future research on NSCLC therapy was presented. This article aimed to provide an overview of the progress of clinical research on TIME and the treatment of NSCLC, which may provide insights for future research to improve the treatment effect by targeting the TIME.

## TIME and immunoediting

The TME is composed of vascular and lymphatic networks, fibroblasts, immune cells, the extracellular matrix, and signaling molecules inside and around the tumor. Tumor cells have been shown to interact closely with the surrounding microenvironment [9]. Local chronic inflammation or exposure to carcinogens may promote the development of cancer. Moreover, tumor cells promote angiogenesis [10], epithelial-mesenchymal transition [11], and extracellular matrix remodeling [12] by changing the local metabolic environment and/or releasing extracellular signals to facilitate their survival and development in the body. The components of immune cells play a vital role in this interaction. Two opposing and dynamic immune processes occur in the TIME: immuno-surveillance and tumor immune escape, which are collectively called immunoediting. The immuno-surveillance process identifies and eliminates nascent tumor cells, whereas the immune escape process allows the tumor to progress [13].

The concept of tumor immunosurveillance has been widely accepted and discussed in detail in several reviews [14-16]. The recognition of tumor-associated antigens (TAAs) by immune cells is the starting point of immunosurveillance. TAAs can be divided into three types. The first type is encoded by normal genes that are expressed in the early developmental germline, placenta, or immune-privileged sites (testis), which include melanoma-associated antigen 1, α-fetoprotein, carcino-embryonic antigen, and cancer testis antigen. The second type is viral antigens, such as Epstein-Barr virus-related antigens. The third type is neoantigens, which are new peptides produced by mutated genes in tumor cells.

CD8^+^ T cells, also known as cytotoxic T lymphocytes (CTLs), are the primary immune cells responsible for destroying tumor cells, whereas CD4^+^ T cells help B cells produce antibodies and perform other immune regulatory functions. T-cell activation involves three signaling processes. Signal 1 is based on the interaction of T-cell receptors (TCRs) with TAAs. TCRs on the surface of CD8^+^ T cells interact with TAAs presented by major histocompatibility complex (MHC) class I molecules on the surface of tumor cells, while TCRs on the surface of CD4^+^ T cells interact with TAAs presented by MHC class II molecules on the surface of antigen-presenting cells (APCs, such as dendritic cells, macrophages, and B cells). Signal 2 is a positive co-stimulatory signal cluster (such as the interaction of CD28 and CD80/86) between T cells and APCs. Signal 3 is the secretion of cytokines such as interleukin-2 (IL-2) from T cells, which triggers the proliferation, differentiation, and survival of T cells targeting specific TAAs. The proliferation of activated T cells occurs in the draining lymph nodes or tertiary lymphatic structures of the tumor [17]. After activation, they migrate into the blood vessels or tertiary lymphatic structures, infiltrate the tumor, and then identify and destroy the tumor cells.

In addition to T cells, innate immunity plays an essential role in immunosurveillance. Several molecules (such as NKG2D) expressed in tumor cells directly bind to the receptors on innate immune cells (such as natural killer (NK) cells) to activate NK cells and destroy tumor cells. Tumor cell death triggers inflammation, which attracts additional innate immune cells, T cells, and B cells [14, 18, 19].

The process by which tumor cells escape immunosurveillance is considered a natural (Darwinian) selection process. Over the past few decades, it has become the focus of tumor immunology research [20]. This immune escape process is diverse and involves both tumor cells and other related immune suppressor cells, including regulatory T cells (Tregs), tumor-associated macrophages (TAMs), myeloid-derived suppressor cells (MDSCs), and cancer-associated fibroblasts (CAFs). Some tumor cells have a low mutation rate or lack qualified TAAs, whereas other tumor cells downregulate or suppress the expression of MHC I molecules, which inhibits the signal 1-based activation of CD8^+^ T cells. In addition, some tumor cells may express inhibitory molecules, such as programmed cell death ligand 1 (PD-L1, an immune checkpoint ligand), which binds to its receptor, PD-1, and inhibits the signal 2-based activation of CD8^+^ T cells. Tumor cells also secrete a variety of inhibitory molecules such as transforming growth factor-β (TGF-β), IL-6, prostaglandin E2, chemokine ligand 2, and colony-stimulating factor 1, which may directly inhibit the expression of CD8^+^ T cells or promote the accumulation of various immunosuppressive cells. Tregs express inhibitory molecules, such as cytotoxic T lymphocyte antigen 4 (CTLA-4, an immune checkpoint molecule), which binds to CD80/CD86 on the surface of APCs to inhibit the activation of signal 2, and they express CD25 and FoxP3 to deplete the levels of IL-2 in the local environment, thereby reducing the activation of signal 3.

Tumor cells also produce other immunosuppressive cytokines, such as TGF-β and IL-10. In addition, TAMs express human leukocyte antigen-G and E (HLA-G and HLA-E), which inhibit MHC-1 expression, thereby blocking signal 1 activation. HLA-E also binds to NKG2 on NK cells to inhibit the activation of NK cells. TAMs also express PD-L1 and CD80/CD86 to block the activation of signal 2 and produce CC chemokine ligands in order to attract more Tregs. Both MDSCs and CAFs inhibit immunosurveillance in the TME through various mechanisms [21, 22]. T-cell exhaustion occurs when effector T cells are exposed to antigens for a long time (during chronic viral infection or when malignant tumors are present). This can be attributed to multiple mechanisms. PD-1 (an immune checkpoint receptor) is mainly expressed on exhausted T cells and binds to PD-L1 to inhibit signal 2 activation [23, 24].

The transition from balanced to imbalanced immune surveillance and immune escape can be divided into three stages. The first stage is called the elimination phase, in which immunosurveillance is predominant. As nascent tumor cells are quickly eliminated, they rarely survive during this phase. Second, in the equilibrium phase, new mutant tumor cells begin to appear, attempting to eliminate the pressure of immune surveillance; however, this process may take a long time. Third, in the escape phase, some mutant tumor cells under selective pressure successfully escape immunosurveillance and proliferate in the body in an uncontrolled manner. The third phase is most observed in clinical practice. The immune cells found in the TIME include T cells, B cells, NK cells, natural killer T (NKT) cells, and TAMs, of which T cells usually account for the majority. However, as tumor progression continues, the activation of effector cells decreases, and immunosuppressive cells dominate [14, 25].

## Cancer immunotherapy

In recent years, immunotherapy, which promotes immunosurveillance and inhibits tumor immune escape, has achieved unprecedented clinical success as a cancer treatment. The 2018 Nobel Prize in Physiology or Medicine was awarded to two immunologists, James P. Allison and Tasuku Honjo, “for their discovery of cancer therapy by inhibition of negative immune regulation”. Several detailed review articles on cancer immunotherapy have been published. Here, we provide a summary of the types of immunotherapies that have been used in clinical practice.

Immune checkpoint inhibitors (ICIs) invigorate the exhausted tumor-infiltrating lymphocytes (TILs) and are the most widely used type of immunotherapy. Since the approval of the first ICI for the treatment of melanoma in 2011 (the anti-CTLA-4 antibody ipilimumab) [26], several monoclonal antibody drugs targeting PD-1 and its ligand PD-L1 have been approved for clinical use and have proven effective for various types of cancer.

Adoptive cell transfer therapy (ACT) uses selected autologous TILs or genetically engineered T cells with chimeric antigen receptors (CARs). After in vitro expansion, they are delivered intravenously to the patient to specifically identify and destroy the tumor cells. To date, five CAR T-cell therapies have been approved by the United States (US) Food and Drug Administration (FDA). Tisagenlecleucel and axicabtagene ciloleucel are suitable for treating B-cell non-Hodgkin’s lymphoma and/or B-cell acute lymphoblastic leukemia (ALL). Brexucabtagene autoleucel is used to treat relapsed/refractory (R/R) mantle cell lymphoma, axicabtagene ciloleucel is indicated for the treatment of adult R/R follicular lymphoma, and lisocabtagene maraleucel is indicated for the treatment of adult R/R large B-cell lymphoma. ACT has not yet showed satisfactory results in the treatment of solid tumors.

The use of therapeutic vaccines, derived from the patient’s own tumor cells, TAAs of certain types of tumor cells, or the patient’s dendritic cells, which enhance the ability of the patient’s immune system to respond to TAAs on the tumor cells, is an active area of immunotherapy research investigation. The US FDA has approved the use of dendritic cell vaccine sipuleucel-T for the treatment of certain advanced-stage prostate cancers. Another treatment strategy similar to therapeutic vaccines is oncolytic virus therapy, which uses an oncolytic virus to selectively infect and destroy tumor cells without harming the normal cells. The destruction of tumor cells stimulates the immune system to fight the remaining tumor. The first oncolytic virus therapy approved by the US FDA was talimogene laherparepvec, which is prepared from herpes simplex virus type 1 and is injected directly in the tumors to treat unresectable melanoma.

Monoclonal antibodies (mAbs) are a type of passive immunotherapy. Certain monoclonal antibodies “tag” the TAAs of tumor cells and direct the immune system to recognize and destroy these cells. For example, rituximab interacts with B cells and certain types of tumor cells. It binds to the CD20 protein to treat B-cell non-Hodgkin’s lymphoma. Other mAbs can help T cells home in on tumor cells to facilitate T-cell-mediated destruction. One example is blinatumomab, which binds to both CD19 on the surface of leukemia cells and CD3 on the surface of T cells, bringing the two cells into close proximity and allowing T cells to destroy the leukemia cells. mAbs have also been conjugated with chemotherapeutic drugs, such as brentuximab vedotin, ado-trastuzumab emtansine, and ibritumomab tiuxetan, or with radioactive particles to help these toxic substances specifically bind to tumor cells; however, these therapies are not considered pure immunotherapy.

Immunomodulators enhance the immune response of tumor cells. Commonly used cytokines include interferon (IFN)-α and IL-2. Biological response modifiers that stimulate the immune system have also been developed; thalidomide, lenalidomide, and pomalidomide cause immune cells to release IL-2 and inhibit the formation of new blood vessels in tumors (www.cancer.gov/, www.cancer.org/) [27].

## TIME research methods for NSCLC

Immunohistochemistry is the most commonly used method to study the TIME in solid tumors, including NSCLC. This method allows the direct observation of the expression of specific antigens in both tumor and immune cells. Some previous studies have used stereotactic methods to analyze the distribution of antigens at different spatial locations in the tumor and surrounding tissues [28, 29]. Paraffin-embedded samples can be stored for many years and are particularly suitable for retrospective analyses [30]. However, the types of immune cells that can be observed are limited, quantification remains challenging, and the location from which the sampled tissue specimen was collected can exert a significant effect on the result. Multiplex immunofluorescence technology is an upgraded version of the traditional immunofluorescence technology. This technology uses tyramide-based or cyclic immunofluorescence markers to perform immunostaining for multiple factors in a single tissue section followed by digital scanning and image processing. The spatial distribution of multiple antigens and their correlations can be obtained simultaneously [31].

Flow cytometry is also widely used to study TIME. This method also uses immunofluorescence staining to identify markers on cells in suspension, followed by quantitative analysis such as cell counting. Because NSCLC is a solid tumor, the tumor tissue must be mechanically separated with scissors or blades and treated with collagenase or other proteases in order to produce single-cell suspensions. Additional preparation steps, such as filtration and centrifugation, must be completed prior to the flow cytometry analysis. This method facilitates the classification and counting of a large number of cells; however, the different processing steps can destroy a portion of the cells from the solid tumor tissue, which can affect the results, and the spatial information of the cells in the tumor tissues can no longer be obtained. Comprehensive observations of the immune cell composition of NSCLC have been conducted using this method [32].

Mass cytometry, or cytometry by time of flight (CyTOF), is a variation of flow cytometry. Compared with standard flow cytometry, antibodies are labeled with unique stable heavy metal isotopes instead of fluorescent dyes during CyTOF. The immunolabeled cells are vaporized in a nebulizer, and the number of heavy metal isotopes is reported through the mass channel as the antigen molecule expression level. As virtually no signal overlap is observed between different heavy metal isotopes, more than 40 cell parameters can be measured simultaneously in a single cell [33, 34]. Based on the same principle, antibodies labeled with unique metal isotopes have also been used for immunostaining of formalin-fixed paraffin-embedded tissue sections, while multiplexed ion beam imaging technology has been used to obtain the imaging data, which could simultaneously detect more than 40 markers at subcellular resolution [35]. Matrix-assisted laser desorption/ionization mass spectrometry imaging has also been used to study the TIME of NSCLC [36].

Single-cell transcriptomics is utilized to isolate a single cell from a tissue and simultaneously measure the mRNA levels of hundreds or thousands of genes in a single cell. Human whole-genome gene expression microarrays and custom microarrays are commonly performed to obtain the gene expression profiles. Typically, hundreds or even thousands of cells are analyzed individually. The analysis of such large numbers of single cells allows the collection of the accurate immunoprofile of the TIME [37]. Although bulk tumor tissue transcriptomic approaches are more commonly used to study TIME, these methods may mask the gene expression information from cells that comprise a smaller proportion of the tumor tissue. Through immunoprofile analysis, prognosis-related gene signatures can be identified, or a TIME scoring system can be established [38, 39].

In recent years, owing to the rapid development of gene sequencing technology, RNA sequencing (RNA-seq) of tumor tissues has been widely used to obtain the gene expression profiles. Unlike microarrays, RNA-seq detects not only annotated transcripts, but also new sequences and splice variants, thus ensuring that more comprehensive transcription information is obtained. Therefore, RNA-seq is more widely used compared with microarrays [40]. Many studies have used this method to explore the mechanisms underlying the interactions among cancer cells, immune subgroups, and matrix components in NSCLC [41, 42]. Single-cell RNA sequencing (scRNA-seq) can provide the gene expression profile of a single cell; therefore, this method is particularly suitable for studying the gene expression pattern of a specific type of cell. Compared with studies of mixed RNA samples extracted from bulk tumor tissues composed of different cells, this method has significant advantages [43]. For example, Guo et al. used scRNA-seq to analyze the global characteristics of T cells in patients with NSCLC [44]. Zhong et al. analyzed the single-cell RNA sequencing data from 11,485 NSCLC cells and revealed the immune cell infiltration in tumor tissues and related marker genes [45].

The TIME status is closely related to the treatment effect. Therefore, the classification has clinical significance. Previously, TIME profiles were divided into four categories based on the immunohistochemistry results: type I (PD-L1 negative and TIL negative, indicating immune ignorance), type II (PD-L1 positive and TIL positive, indicating adaptive immune resistance), type III (PD-L1 negative and TIL positive, implying the role of other inhibitors in promoting immune tolerance), and type IV (PD-L1 positive and TIL negative, indicating intrinsic induction) [46, 47].

The phenotypes of the TIME could be classified into three major categories based on the medium-resolution data established from low-resolution sources (e.g., bulk tissue microarrays and immunohistochemistry) using bioinformatics technology; more subcategories could be further distinguished with the application of high-resolution technologies (such as scRNA-seq, flow cytometry, and imaging technology). The first category is called infiltrated-excluded TIME, which is characterized by the absence of CTLs in the core of the tumor. CTLs exist at the edge of the tumor, where they are in contact with TAMs or located in the fibrotic nests. The second category is called infiltrated-inflamed (I-I) TIME, which is characterized by presence of numerous CTLs expressing PD-1 infiltrating the interior of the tumor, leukocytes, and tumor cells expressing PD-L1. The third category is a subcategory of the I-I TIME, called the tertiary lymphoid structure (TLS) TIME, which is characterized by the presence of TLSs in the tumor margins or stroma, similar to the lymph nodes, and is composed of immune cell aggregates, including B cells, dendritic cells, and Treg cells [48].

## Relevance of the TIME for the treatment of NSCLC

Growing evidence shows that the TIME status in patients with NSCLC can predict their prognosis after treatment, and certain treatments may significantly improve the TIME, which makes it conducive for further combination treatment [49-52]. Clinical studies have more direct significance in guiding clinical practice than in vitro or animal research; hence, the next section discusses clinical research on the interaction between TIME and treatments for NSCLC.

### Chemotherapy and the TIME in NSCLC

One of the postulated mechanisms by which chemotherapy exerts its antitumor effects is the immunosuppressive response. However, several chemotherapeutic drugs (such as paclitaxel, cisplatin, gemcitabine, and carboplatin) cause tumor cell death and TAA release, thereby activating APCs and, subsequently, the immune response [53].

A randomized trial compared the efficacy of induction chemotherapy followed by surgery with surgery alone in patients with stage IIIA N2 NSCLC. In the 122 patients, 21 pairs of lymph nodes were collected before and after neoadjuvant chemotherapy (NAC). No significant differences were observed in the PD-L1 expression after comparing the specimens before and after the treatment. When the tumors from patients receiving NAC were compared with those from patients who did not receive chemotherapy, no difference was detected between the number of tumor-infiltrating CD8^+^ T cells and DC-LAMP^+^ cells (mature dendritic cells). No significant correlation was observed between PD-L1 expression in tumors or immune cells and the clinical outcomes of patients; however, a high density of CD8+ T cells and DC-LAMP^+^ cells was associated with better clinical outcomes. Moreover, the densities of CD8^+^ and DC-LAMP^+^ cells were positively correlated with the expression of PD-L1 on tumor cells. Thus, the combination of conventional chemotherapy and immunotherapy may improve therapeutic effects [54].

In 92 patients with NSCLC, the PD-L1 expression increased in chemoresistant tumors compared with that in chemotherapy-sensitive tumors, and the rate of PD-L1-positive staining was correlated with TNM stage, lower treatment response rate, and shorter overall survival rate. Additionally, patients with high PD-L1 expression who received chemotherapy have a poorer prognosis. CD8^+^ TIL count was associated with chemotherapy sensitivity, better prognosis, and decreased PD-L1 expression. High PD-L1 expression after NAC might indicate treatment resistance and poor prognosis in patients with NSCLC. Therefore, PD-L1-targeted therapy may be a promising strategy for reversing lung cancer chemoresistance [55].

According to the results of immunohistochemical analysis, the expression of PD-L1 on NSCLC cells tended to decrease in patients receiving tyrosine kinase inhibitors (TKIs) or taxane therapy but not in those receiving pemetrexed-based therapy. No apparent changes were observed in the TILs. In addition, the PD-L1 expression in tumor cells was significantly reduced in patients who responded to NAC but not in those who did not receive NAC. Therefore, PD-L1 expression should be closely monitored when using PD-1 inhibitors [56].

Parra et al. compared the immunological markers in resected tumor tissues of 112 patients who did not receive NAC and in those of 51 patients with stage II/III NSCLC who received NAC. The PD-L1 expression and overall density of tumor-associated immune cells, CD68^+^ TAMs, epithelial and stromal helper T cells (CD3^+^CD4^+^) and activated natural killer cells (CD57^+^ granzyme B CD45RO^-^) were higher in the epithelial and stromal compartments of patients who received NAC than in those who did not receive NAC. In patients who received NAC, higher numbers of epithelial helper T cells and epithelial and stromal TAMs were associated with better prognosis. Therefore, NAC may activate the immune response in lung cancer patients, which may improve the efficacy of subsequent ICI therapy [57].

The changes in PD-L1 expression and CD8^+^ TIL density were also investigated in biopsy tissues collected both before and after tumor recurrence in 17 patients who received platinum-based adjuvant chemotherapy or chemotherapy as treatment for an advanced stage tumor. Compared with the original biopsy sample, the expression of PD-L1 tended to increase in the re-biopsied sample, but this difference was not significant; moreover, the density of CD8^+^ TILs did not increase [58].

The number of CD8^+^ cells, expression of functional markers (IFN, granzyme B, and perforin), and levels of specific chemokines in tumor samples from 100 patients with NSCLC were analyzed [59]. Compared with oxaliplatin alone (n=72), docetaxel combined with oxaliplatin treatment (n=5) increased the intratumor CXCL11 expression by approximately 10 times, and the number of CD8^+^ TILs also increased significantly. When the preoperative diagnostic puncture specimens and postoperative specimens obtained after docetaxel chemotherapy were compared, the number of CD8^+^ TILs significantly increased in postoperative specimens. The colocalization of CD8 and CXCR3 in lung tumor tissues was also observed. In addition, the increase in the CD8^+^ TIL count and upregulation of its functional markers IFN-α, perforin, and granzyme B, as well as HMGB1 and CXCL11, were positively correlated with overall survival [59].

Fournel et al. evaluated the effect of cisplatin treatment on PD-L1 expression by analyzing the matched tumor tissues obtained from 39 patients with NSCLC before and after chemotherapy. Cisplatin treatment significantly increased the PD-L1 expression in tumors and immune cells, but had no significant effect on the CD8^+^ lymphocyte count; moreover, a significant correlation was not observed between PD-L1 expression in tumor cells and the corresponding CD8^+^ lymphocytes. Because cisplatin treatment increases the PD-L1 expression, it could exert a synergistic effect when combined with PD-1 inhibitors [60].

PD-L1 expression and CD8^+^ TILs count in primary tumor specimens obtained from surgical resection and re-biopsied specimens of 36 patients with NSCLC who had recurrent tumors were compared in the study by Shimizu et al. Results showed absence of changes in the PD-L1 expression (77.8%) or CD8^+^ TIL counts (72.2%) in most patients. However, a greater proportion of patients who received platinum-based chemotherapy showed an increased PD-L1 expression in their biopsy specimens. In addition, a lower proportion of patients who received tegafur chemotherapy had decreased CD8^+^ TIL counts (36.4%) in their biopsy specimens. Based on these results, chemotherapy might increase the PD-L1 expression. For patients with recurrent NSCLC, especially those who have previously received chemotherapy, a biopsy is required to determine whether PD-1/PD-L1 inhibitors should be used as a treatment [61].

To evaluate the effect of conventional first-line platinum-based chemotherapy on the TIME, the expression of 201 cancer- and immune-related genes in matched samples collected from 29 patients with NSCLC before and after chemotherapy was investigated. An immune co-expression cluster, including PD-1 and PD-L1, and three other co-expression clusters were also identified. Platinum-based chemotherapy reduced the average gene expression in the immune cluster but had no significant effect on the other three clusters. In this immune cluster, the expression levels of CTLA4, LAG3, TNFRSF18, CD80, and FOXP3 mRNA were significantly decreased; therefore, traditional platinum-based chemotherapy exerts a negative effect on the TIME [62].

Recently, Gaudreau et al. reported the effect of NAC on TIME in patients with resectable NSCLC. They found that NAC increased the infiltration of CD8^+^ cytotoxic T cells and CD2^+^ B cells and increased the numbers of CD8^+^ CD103^+^ and CD4^+^ CD103^+^ PD1^+^ TIM3^-^ tissue-resident memory T cells. These data suggest that NAC could improve the antitumor immunity by recruiting T and B cells and promoting cytotoxic and memory CD8^+^ T-cell and CD4^+^ memory helper T-cell phenotypic trans-formation [63].

The TLS in patients with NSCLC is associated with prolonged survival [64]. Siliņa et al. analyzed the formation of TLSs in samples of lung squamous cell carcinoma obtained from 138 patients with sequential stages of TLS maturation and the final germinal center. In untreated patients, a high TLS density was significantly associated with upregulated expression of adaptive immune-related genes, including CD27, CD8A, IL21, and IGKC, and prolonged progression-free, disease-free, and overall survival times. The expression of most TLS-related genes was associated with the improvement in progression-free survival. In patients treated with NAC, the TLS density was similar, but the germinal center formation was impaired, and the prognostic value of TLS density disappeared. Corticosteroids combined with chemotherapy have been shown to control the side effects in patients with cancer. Among patients with lung squamous cell carcinoma who had not received chemotherapy, the preoperative TLS density and germinal center formation were reduced in those receiving glucocorticoid treatment, and the negative effect of corticosteroids on TLS formation was associated with poor prognosis in patients who received chemotherapy alone. Therefore, the treatment plans should incorporate nonsteroidal alternative drugs to ameliorate the side effects of chemotherapy, radiotherapy, and immuno-therapy [65].

Results of the multiple clinical studies mentioned above indicate that chemotherapy either has no effect on PD-L1 expression in NSCLC cells or upregulates its expression, especially in chemoresistant tumors and tumors that relapse after chemotherapy. PD-L1 expression was also upregulated. Therefore, many studies have suggested the use of chemotherapy in combination with ICIs, but the most appropriate approach is to assess the PD-L1 expression level before using these drugs. However, the effects of chemotherapy on the number of CD8^+^ TILs and other immune responses remain unclear. Some studies found that the average gene expression of the immune cluster decreased upon chemotherapy, others did not report significant changes, and others found that chemotherapy improved the antitumor immune response. However, all studies clearly showed that a high density of CD8^+^ TILs in tumor samples prior to treatment is associated with better clinical outcomes. In addition, one study showed that the use of glucocorticoids to control the side effects of chemotherapy might significantly inhibit the antitumor immunity. Hence, alternative nonsteroidal drugs are recommended to ameliorate these side effects.

### ICI therapy and the TIME in NSCLC

The assessment of PD-L1 expression in tumor cells using immunohistochemistry has become a standard diagnostic method for patients with NSCLC treated with a single ICI [66]. Mazzaschi et al. retrospectively analyzed 100 patients with stage I to IIIa NSCLC who had undergone primary tumor resection without neoadjuvant therapy and 26 patients with advanced NSCLC who were treated with nivolumab. Patients with a high number of CD8^+^ lymphocytes and a lack of PD-1 inhibitory receptors or a low PD-1 to CD8 ratio had longer progression-free survival and overall survival times and responded better to nivolumab treatment. The levels of PD-L1 expression were higher in CD8^+^ TILs from NSCLC samples. In addition, compared with wild-type tumors, the numbers of CD8^+^ and PD-1^+^ cells in EGFR-mutant tumors were significantly reduced, but the PD-L1 expression remained unchanged. However, the number of PD-1^+^ TILs and PD-L1 expression decreased in KRAS-mutant tumors. Therefore, patients with high CD8^+^ lymphocyte counts, and a lack of PD-1 inhibitory receptors have a good prognosis [67].

A subset of CD25^+^CD4^+^ T cells with higher expression of PD-L1 (PD-L1 Tregs) were detected in 42 surgically resected NSCLC specimens, and the frequency of PD-L1 Tregs positively correlated with the number of CD8^+^ TILs that expressed PD-1. Patients with high expression levels of both PD-1^+^CD8^+^ TILs and PD-L1 Tregs in the TIME showed “greater PD-1/PD-L1 pathway-dependent CD8^+^ T-cell exhaustion.” They also analyzed 31 patients receiving PD-1 blocking immunotherapy and found that those with high densities of PD-1 CD8^+^ TILs and PD-L1 Tregs responded better to treatment [68].

Thommen et al. investigated the transcriptional, metabolic, and functional characteristics of CD8^+^ T lymphocytes in tumors from patients with NSCLC. Compared with TILs with intermediate or no PD-1 expression, TILs with high PD-1 expression had higher production of inhibitory receptors, showed loss of effector cytokine secretion, were susceptible to immuno-suppressive factors (such as IL-10), and had an inherently high tumor recognition ability. When this high PD-1 TIL population was expanded in vitro, the PD-1 expression, metabolism, and function returned to normal. In patients with NSCLC treated with PD-1 blockers, the presence of a large number of PD-1 TILs strongly predicted an increase in overall survival and a durable treatment response [69].

A pooled analysis of 938 patients with NSCLC treated with atezolizumab (anti-PD-L1) was conducted by Kowanetz et al. Results showed that high PD-L1 expression in tumor cells alone or immune cells alone was associated with a durable clinical response to atezolizumab treatment. In tumor tissues collected ~6 months after the first administration of atezolizumab, the CD8^+^ T-cell infiltration in the intraepithelial area significantly increased, accompanied by increased expression of T effector cell markers (IFNG, GZMB, and PRF1) and T-cell chemokines (CXCL9 and CXCL10). Therefore, in patients with NSCLC, the expression of PD-L1 on tumor cells and infiltrating immune cells might predict the therapeutic response to atezolizumab and plays a non-redundant role in regulating antitumor immunity [70].

A recent study showed that a high density of CD3^+^ and CD8^+^ TILs was associated with a better treatment response, based on a study of 15 patients with advanced NSCLC treated with nivolumab. The densities of CD4^+^, CD57^+^ NK, GrzB^+^ lymphocytes, and CD68^+^ TAMs were not significantly related to treatment response. PD-L1 expression was not also significantly related to treatment response, but low PD-1 expression in CD8^+^ TILs was closely correlated with better treatment response [71].

The predictive roles of PD-L1 and CD8 expression in patients with stage IIIB or IV NSCLC treated with nivolumab after failure of platinum-based chemotherapy were evaluated by Fumet et al. Seventy-eight samples were subjected to immunohistochemical analysis, while 43 samples were subjected to RNA-seq. High CD8^+^ TIL counts and CD8A (CD8 gene) expression levels were significantly correlated with a higher response rate and longer progression-free survival. PD-L1 expression was not related to the response rate, but high CD274 (PD-L1 gene) expression was associated with longer progression-free survival. Patients with high CD8^+^ TIL counts and PD-L1 expression levels detected using immuno-histochemistry or high CD8A and CD274 co-expression had a longer progression-free survival [72].

The combination of PD-1 inhibitors and chemotherapeutic drugs enhances the efficacy of NSCLC treatment, but the best combination protocol and underlying mechanism remain uncertain. Ogawara et al. compared three treatment options: salvage chemotherapy after nivolumab, nivolumab alone, and chemotherapy before nivolumab. Salvage chemotherapy after nivolumab treatment significantly enhanced the antitumor activity, and the response to salvage chemotherapy was not associated with the expression of PD-L1 in tumor cells. In addition, fewer PD-1^+^ immune cells were detected in responders than in nonresponders. High PD-1-expressing CD8^+^ immune cells might be deeply exhausted from PD-1 inhibitor treatment and irreversibly lose their function. This treatment option requires further study [73].

Another study analyzed 30 patients with stage IV NSCLC who received nivolumab monotherapy and found that high tumor mutational load (TML) was significantly associated with prolonged progression-free survival and overall survival. Results of the interaction analysis showed that patients with both high TML and high total CD8^+^ T-cell infiltration rates, high TML and no HLA class-I loss, high total CD8^+^ T-cell infiltration and no HLA class-I loss, high PD-L1 expression levels and high TML, and high PD-L1 expression levels and no HLA class I loss had significantly longer progression-free survival. Therefore, PD-L1 expression, TML, CD8^+^ T-cell infiltration, and HLA-type I expression are recognized as biomarkers for predicting the effects of ICI treatment [74].

PD-L1 expression and immune cell infiltration in 139 patients with advanced NSCLC treated with nivolumab were evaluated by Fumet et al. Tissue blocks were obtained before or after the last line of platinum doublet chemotherapy, before ICI treatment. TIME examination of the biopsy tissue after the last-line chemotherapy predicted a durable clinical benefit and favorable overall survival, whereas the tissue specimen examined before last-line chemotherapy did not. Moreover, a high level of infiltration of stromal CD8^+^ immune cells, stromal CD4^+^ immune cells, tumor and stromal CD8^+^ immune cells, and PD-1^+^ and CD8^+^ immune cells was associated with longer overall survival. This prolonged survival outcome indicates that conventional chemotherapy might change the TIME, thereby affecting the subsequent treatment effect of ICIs [75].

Jang et al. conducted an unsupervised clustering analysis of mRNA sequencing data from 87 lung adenocarcinoma and 101 lung squamous cell carcinoma specimens, and evaluated the molecular subtypes based on the immunogenomic determinants in the transcriptomes. The correlation between molecular subtype and treatment response in 35 patients with NSCLC treated with PD-1 inhibitors was analyzed, and the results showed two different subtypes of NSCLC tumors. The immunogenic subtype had higher expression of immunomodulatory molecules, higher cytolytic activity, lower tumor purity, and a higher proportion of CD8^+^ T cells and memory B cells. However, the proportion of regulatory CD4^+^ T cells and tumor-related bone marrow cells was lower. This subtype responded well to PD-1 inhibitor therapy.

By contrast, the immune-resistant subtype had a lower expression of immunomodulatory and MHC molecules and a lower proportion of CD8^+^ T cells, memory B cells, and CD4^+^ T cells; however, the levels of regulatory CD4^+^ T cells, tumor-promoting macrophages, and tumor-related dendritic cells were higher. This subtype has poor response to PD-1 inhibitor treatment. The molecular subtypes, based on the transcriptome, predicted the response to PD-1 inhibitor treatment. In addition, PD-1 inhibitors block the inhibitory pathway in T cells and eliminate the inhibitory effects of other immune cells, such as B cells [76].

In summary, one study found that ICI therapy increased the CD8^+^ T-cell infiltration. The prognostic value of TIME for ICI treatment is consistent with that of chemotherapy alone. Patients with a high CD8^+^ TIL density in NSCLC tumor tissues before treatment responded better to ICI treatment. High densities of PD-1^+^ and CD8^+^ immune cells are associated with a better treatment response. However, if CD8^+^ TILs also express high levels of PD-1, the therapeutic effect of ICIs is reduced. This change occurs because CD8^+^ immune cells that express PD-1 may be deeply exhausted; thus, the PD-1 inhibitors are unable to restore their function. In addition, some studies have found that PD-L1 expression is not related to the therapeutic effect of ICIs, while others have found that high PD-L1 expression is associated with a better response.

### EGFR TKI therapy and the TIME in NSCLC

EGFR mutations are among the most common driver oncogene mutations in NSCLC. EGFR-TKIs exert a significant effect on patients with advanced NSCLC carrying EGFR mutations and have become the standard first-line treatment for these patients. However, 20%-30% of patients do not respond to EGFR-TKIs [77].

The clinicopathological characteristics and TIME were analyzed in 124 patients with NSCLC carrying EGFR mutations who received EGFR-TKIs. Low total TIL counts (CD4^+^ and CD8^+^ TILs) and negative PD-L1 expression were significantly associated with primary resistance to EGFR-TKIs. These results indicate that primary drug resistance in patients with EGFR mutations is substantially related to immune ignorance. Therefore, a combination therapy, such as anti-CTLA-4 and anti-PD-1 antibodies, is recommended to promote the infiltration of T cells into the tumor and further help maintain its activity [78].

A total of 110 patients with advanced NSCLC carrying EGFR mutations, who were treated with EGFR-TKIs, were investigated. Notably, high PD-L1 expression significantly decreased the objective response rate and progression-free survival. The PD-L1 expression and CD8 double-positive rates were higher in patients with primary drug resistance, indicating that the TIME was activated. Therefore, PD-1 inhibitor therapy is recommended in these patients. One patient received PD-1 inhibitor therapy and showed a favorable response [79].

The efficacy of targeting TIME in 70 patients with advanced EGFR-mutant lung adenocarcinoma has been investigated using TKIs as the first-line treatment. Results showed that patients with high PD-L1 tumor proportions and high CD8^+^ scores had the lowest response rate. The shortest progression-free survival was observed in these patients, and the longest survival was observed in patients with a low proportion of PD-L1 tumors and a high CD8^+^ score. The authors were unable to provide a complete explanation of this result but suggested that patients with NSCLC carrying EGFR mutations should be treated appropriately based on the mutation subtype [80].

Isomoto et al. evaluated the changes in the TIME before and after EGFR TKI treatment in 138 EGFR mutation-positive patients with NSCLC; they found that EGFR TKI treatment caused a significant increase in the expression of PD-L1 and CD73, a cell surface enzyme that is overexpressed in the TIME, inhibits antitumor immunity, and promotes tumor growth [81], especially in T790M-negative patients. After EGFR TKI treatment, the tumor mutational burden (TMB) tended to increase, while the densities of CD8^+^ and FOXP3^+^ TILs significantly decreased; however, the densities of CD8^+^ and FOXP3^+^ TILs in strongly PD-L1-positive tumors were significantly higher than those in PD-L1-negative or PD-L1^low^ tumors. In addition, patients with increased PD-L1 expression in tumor cells after EGFR-TKI treatment responded better to subsequent ICI treatment. The change in the TIME of EGFR-mutant NSCLC caused by EGFR-TKI treatment improved the effect of subsequent ICI treatment [82].

Multiplex immunohistochemical staining was performed to examine the correlation between PD-L1 expression and TIME in 15 patients with stage III or IV lung adenocarcinoma carrying EGFR mutations and their response to EGFR TKI treatment. PD-L1 expression was negatively correlated with CD20^+^ B cells, CD68^+^ macrophages, and Tregs and that a lack of B-cell infiltration was related to a poor TKI response. Future studies should examine the effect of B cells in the TIME of patients receiving EGFR-TKI treatment [83].

In summary, a previous study found that EGFR TKI treatment increases the expression of PD-L1 in NSCLC patients with EGFR mutations and decreased the density of CD8^+^ and FOXP3^+^ TILs. Among patients receiving EGFR-TKI treatment, those with increased PD-L1 expression responded better to subsequent ICI treatment. Previous studies have found that primary resistance to EGFR-TKIs is related to low total TIL counts (CD4^+^ and CD8^+^ TILs), high PD-L1 expression, and high PD-L1 and CD8 double positivity. Another study reported that a lack of B-cell infiltration was related to a poor TKI response. Therefore, combined ICI therapy should be used in this patient group after receiving EGFR-TKI therapy.

### ICI therapy and the TIME in NSCLC patients with mutations in EGFR or other genes

The effects of treatment on 28 EGFR mutation- or ALK-positive patients and 30 EGFR wild-type/ALK-negative patients with NSCLC, as well as the PD-L1 expression and density of CD8^+^ TILs in the TIME, were retrospectively evaluated. Immunohistochemistry was performed to determine the response to PD-1/PD-L1 inhibitor treatment of different clinically relevant molecular subgroups of patients with NSCLC. Patients with EGFR mutations and ALK^+^ lung cancer had reduced response to PD-1/PD-L1 inhibitors. Only a small proportion of patients with EGFR mutation-positive and ALK-positive NSCLC presented both high levels of PD-L1 expression and high density of CD8^+^ TILs in their tumor tissues. The lack of CD8^+^ immune effector cells in the TIME explains why PD-1/PD-L1 inhibitors are ineffective despite the expression of PD-L1 in these patient populations [84].

The most common cause of drug resistance during EGFR-TKI treatment is the secondary T790M mutation in EGFR [85]. Haratani et al. studied 25 EGFR mutation-positive NSCLC patients treated with the PD-1 inhibitor nivolumab after disease progression during the course of EGFR TKI treatment. PD-1 inhibitors exerted a better effect in T790M-negative patients, and PD-L1 was expressed at higher levels in T790M-negative tumors than in T790M-positive tumors. They also analyzed an independent cohort of 60 EGFR mutation-positive patients with NSCLC who progressed during the course of EGFR-TKI treatment and confirmed that T790M-negative patients had higher PD-L1 expression levels. In addition, no difference was observed in the CD8^+^ TIL density between T790M-positive and T790M-negative patients; however, high PD-L1 expression levels and high CD8^+^ TIL density were found in T790M-negative patients. The CD8^+^ TIL density and non-synonymous mutation burden were significantly higher in patients who responded to PD-1 inhibitors. The authors speculated that due to the high level of PD-L1 expression in the tumors of T790M-negative patients, the response to PD-1 inhibitors was better [86].

Another retrospective study analyzed nine patients with EGFR-mutant NSCLC treated with nivolumab to evaluate the biomarkers that could be used to predict the efficacy of PD-1/PD-L1 inhibitors. Prior to nivolumab treatment, eight patients were treated with EGFR TKI. Unlike wild-type EGFR NSCLC, the expression of PD-L1 in tumor cells was not associated with response to nivolumab treatment, and the treatment response was poor. A high density of CD4^+^ T cells and Foxp3^+^ Tregs, rather than CD8^+^ T cells, has clinical benefits [87].

Chen et al. studied the characteristics of TIME in patients with NSCLC with EGFR or HER2 exon 20 insertion mutations (Ex20ins) and explored their correlation with the therapeutic efficacy of PD-1/PD-L1 blockers. Their study included 1,270 patients with NSCLC, of whom 504 (39.7%) had EGFR mutations, 35 (2.8%) had EGFR Ex20ins, and 21 (1.7%) had HER2 Ex20ins. The PD-L1 expression level in patients with EGFR Ex20ins was significantly higher than that in patients with HER2 Ex20ins. A high PD-L1 expression was found to be significantly related to the therapeutic efficacy of PD-1/PD-L1 inhibitors, whereas no correlation was observed between the number of CD4^+^/CD8^+^ TILs and the prognosis of patients with EGFR- or HER2-mutant NSCLC. Patients with EGFR Ex20ins were sensitive to PD-1/PD-L1 blockade, which may be related to the high PD-L1 expression [88]. Mutations in the Kirsten rat sarcoma viral oncogene homolog (KRAS) gene are the most prevalent driver oncogenes in NSCLC. According to a previous study, 11.2% of lung adenocarcinoma patients in Asian countries and 26.1% in Western countries carry KRAS mutations [89]. Unlike other essential mutation subtypes, inhibitors that are effective against the KRAS mutation subtype are not currently available in routine clinical practice. Liu et al. conducted a systematic pooled analysis of 5,326 patients from 23 studies and found that KRAS-mutant tumors were more likely to be PD-L1 positive compared with KRAS wild-type tumors. They also examined 231 surgically resected specimens using immuno-histochemistry and found that the KRAS mutation group had a higher proportion of strong PD-L1-positive cells, higher CD8^+^ T-cell infiltration, and a significantly higher proportion of PD-L1-CD8^+^ TIL double-positive cells compared with the wild-type group. This result suggests the presence of “an inflammatory phenotype with adaptive immune resistance” in the TIME of KRAS-mutant tumors. They analyzed the mutation data obtained from The Cancer Genome Atlas (TCGA) database and found that patients with KRAS mutations had a higher TMB than those with wild-type KRAS; this finding indicate that KRAS-mutant tumors may produce more neoantigens, which could enhance immunogenicity. They also conducted a systematic analysis of the response to anti-PD-1/PD-L1 immunotherapy in a pool of 1,716 patients from 9 studies and found that the objective response rates of the KRAS-mutant group were significantly higher than those of the KRAS wild-type group. However, animal experiments have indicated that the combination of PD-L1 blockers and docetaxel did not improve the therapeutic effect. Therefore, PD-1/PD-L1 inhibitor monotherapy may be the best treatment option for NSCLC patients with KRAS mutations [90].

Jin et al. performed an integrated analysis using publicly available data to evaluate the correlation between ICI treatment, TIME, and classic driver oncogene mutations in East Asian NSCLC patients. Results suggest that the inferior response to ICIs in patients with EGFR mutations and ALK rearrangement may be related to immune ignorance, as indicated by a higher proportion of PD-L1^-^/TIL^-^ tumors and a lower proportion of PD-L1^+^/TIL^+^ tumors, as well as an enriched resting memory CD4^+^ T-cell population and a lack of activated memory CD4^+^ T cells in these tumors. Patients carrying KRAS mutations respond better to ICIs, which may be related to their inflammatory phenotype and adaptive immune resistance, characterized by a higher proportion of PD-L1^+^/TIL^+^ tumors. In addition, a higher proportion of EGFR L858R-mutant tumors were PD-L1^+^/TIL^+^, suggesting that ICIs may be effective against these tumors [91].

For NSCLC patients carrying EGFR or other mutations, the therapeutic effect of ICIs and their association with TIME are complicated. A previous study found that among patients with EGFR mutations or ALK-positive NSCLC, a lower proportion of patients had PD-L1^+^/TIL^+^ tumors and had poor response to ICI treatment. However, among patients with EGFR L858R-mutant tumors, a higher proportion of patients had PD-L1^+^/TIL^+^ tumors; these patients may respond well to ICI treatment. Another study found that the expression of PD-L1 in NSCLC patients with EGFR mutations was not associated with the response to ICI treatment; a high density of CD4^+^ T cells and Foxp3^+^ Tregs had a clinical benefit. Another study reported that the PD-L1 expression among patients with NSCLC without EGFR T790M mutations was higher compared with that among patients with T790M-positive tumors, and they responded well to PD-1 inhibitors. In addition, PD-L1 expression was relatively high in patients with NSCLC carrying the EGFR Ex20ins, and these patients responded well to PD-1 inhibitors. A higher proportion of patients carrying KRAS mutations had PD-L1^+^/TIL^+^ tumors and responded well to ICI treatment.

### Chemoradiotherapy and the TIME in NSCLC

Chemotherapy, radiotherapy, and chemoradiation therapy trigger changes in the TIME and enhance the efficacy of concurrent or sequential immunotherapy [92]. Most of the patients with locally advanced NSCLC (LA-NSCLC) are inoperable and require multimodal treatment. The efficacy of concurrent chemoradiotherapy was better than that of sequential radiotherapy or radiotherapy alone [93].

Tokito et al. retrospectively investigated the predictive relationship between PD-L1 expression and CD8^+^ TIL density in 74 LA-NSCLC patients who received concurrent radiotherapy and chemotherapy (platinum-containing chemotherapy). Results showed that a high CD8^+^ TIL density was associated with longer progression-free survival and overall survival, while PD-L1 expression was not associated with progression-free survival or overall survival. No correlation was observed between PD-L1 expression and CD8^+^ TIL densities. Subgroup analysis showed that PD-L1^-^/CD8^high^ and PD-L1^+^/CD8^low^ groups had the longest and shortest progression-free survival and overall survival, respectively [94].

Another study analyzed the tumor specimens from 45 patients with advanced NSCLC who received concurrent chemoradiation therapy. Among patients with sufficient paired samples before and after treatment, 45.7% (16/35) exhibited reduced PD-L1 expression after treatment, 42.9% (15/35) showed no changes in the level of PD-L1 expression, and 11.4% (4/35) had elevated PD-L1 expression levels. Approximately 2.9% (1/34) of the patients exhibited a decrease in interstitial CD8^+^ lymphocyte density after treatment; meanwhile, this parameter remained unchanged in 47.1% (16/34) of the patients and increased in 50.0% (17/34) of the patients. The expression of PD-L1 in pretreatment specimens (n=45) and post-treatment specimens (n=35) was not associated with recurrence-free survival. However, a decrease in PD-L1 expression was significantly associated with a longer overall survival. In pre- and post-treatment specimens, patients with high and moderate CD8^+^ lymphocyte densities had prolonged recurrence-free survival compared with those with low CD8^+^ lymphocyte densities. Changes in CD8^+^ lymphocyte density were not associated with the recurrence-free or overall survival. Therefore, concurrent chemoradiation therapy can be combined with PD-1 inhibitors to achieve an optimal effect [95].

In a study by Yoneda et al., tumor PD-L1 expression and the density of stromal CD8^+^ TILs were compared in 23 pairs of NSCLC samples obtained before and after concurrent chemoradiotherapy. Eighteen patients who received drug treatment alone served as the control group. Results showed a significant upregulation of PD-L1 expression in tumor cells after concurrent chemoradiotherapy, which was unrelated to the PD-L1 expression status before treatment. In addition, the stromal CD8^+^ TIL density increased significantly after concurrent chemoradiotherapy, which was significantly correlated with better prognosis [96].

Another retrospective study evaluated the prognostic value of PD-L1 expression and CD8^+^ TIL density in initial tumor biopsy samples from 31 patients with inoperable LA-NSCLC who received concurrent chemo-radiotherapy. Results showed that low PD-L1 expression in tumor cells is associated with prolonged overall survival, prolonged progression-free survival, and improved local control. Low CD8^+^ TIL density tended to be associated with longer overall survival and better local control, and CD8^+^ TIL density was positively correlated with PD-L1 expression. Subgroup analysis showed that the PD-L1^-^/CD8^low^ group and PD-L1^+^/CD8^low^ group had the longest and shortest overall survival, respectively [97].

Shirasawa et al. studied the effect of chemoradiotherapy on the TIME of patients with unresectable LA-NSCLC and the effect of anti-PD-L1 therapy in patients who experienced relapse after chemoradiotherapy treatment. The PD-L1 expression did not specifically change in these patients after chemoradiotherapy, but the density of CD8^+^ TILs increased. Anti-PD-L1 therapy is effective in patients with LA-NSCLC who experienced relapse after receiving chemoradiotherapy, regardless of the level of PD-L1 expression prior to chemoradiotherapy [98].

In summary, chemoradiotherapy increases the density of CD8^+^ lymphocytes, which is significantly associated with better prognosis. However, the effects of chemoradiotherapy on PD-L1 expression are inconsistent. Some studies have found that chemoradiotherapy upregulates PD-L1 expression, whereas other studies have not reported a pronounced effect. Thus, the prognostic value of TIME in chemoradiotherapy remains unclear. Some studies have reported that high CD8^+^ TIL density is associated with longer progression-free survival and overall survival, and patients with PD-L1^-^/CD8^high^-level tumors had the longest overall survival. However, another study revealed that low CD8^+^ TIL density prolongs the overall survival and that patients with PD-L1^-^/CD8^low^-level tumors had the longest overall survival. In most studies, PD-L1 expression was not associated with progression-free survival or overall survival.

### Regional treatment and the TIME in NSCLC

Studies have shown that high-dose radiation therapy may lead to lymphopenia, overall immunosuppression, and poor prognosis. However, in a small number of patients, radiotherapy exerts non-targeting effects: a tumor in one part of the body is irradiated, but tumor regression is observed in other parts of the body that have not been irradiated, or only a part of the tumor is irradiated, but regression is observed in the surrounding tumor tissue that does not receive radiation. Radiation induces the release of TAAs to activate APCs and strengthen immunosurveillance, which manifests clinically as non-targeting effects [99].

Hypofractionated stereotactic radiation therapy (HSRT) is a radiation technology that provides precise targeted high-dose radiation to tumors, while minimizing damage to the surrounding tissues. Chang et al. performed a pooled analysis to compare the efficacy of HSRT and lobectomy in treating operable stage I NSCLC. The overall survival of patients receiving HSRT was longer than that of patients who underwent surgery [100].

Another study analyzed the changes in immune cells in the peripheral blood of patients with NSCLC who underwent HSRT and found that HSRT increased the expression of total T cells, especially CD8^+^ T cells, while the expression of inhibitory Tregs was reduced. HSRT also transforms peripheral CD8^+^ T cells into activated T cells, increasing the expression of TNF-α, IFN-γ, granzyme B, and IL-2. Based on these results, HSRT can be used to activate peripheral immune response [101].

Tubin et al. developed a novel stereotactic body radiotherapy-based partial tumor irradiation of hypoxic clonogenic cells (SBRT-PATHY) to focus radiation on the tumor center while preserving the peritumoral immune microenvironment and regional circulating lymphocytes. They used SBRT-PATHY to treat 20 patients with unresectable NSCLC. Results showed that compared with the recommended standard chemotherapy and conventional palliative radiotherapy groups, BRT-PATHY improved the treatment effect and minimized the side effects [102].

Invariable NKT (iNKT) cells play an important role in tumor immunity. iNKT cells are activated by the specific glycolipid antigen α-galactosylceramide (α-GalCer) and exhibit antitumor activity through the production of IFN-γ. Nagato et al. treated four operable patients via an intravenous injection of α-GalCer-pulsed APC prior to surgery for advanced NSCLC. The number of iNKT cells among TILs and the amount of IFN-γ produced by α-GalCer-stimulated TILs in the removed tumors significantly increased [103]. Moreover, Ishibashi et al. conducted a phase I study on transbronchial injection of α-GalCer-pulsed APC in 21 patients with advanced or recurrent NSCLC who were refractory to standard therapies. One patient showed a partial response, while eight achieved stable disease. In addition, the iNKT count in the peripheral blood mononuclear cells increased in eight patients. In the TIME, IFN-γ expression was upregulated after treatment in all nine patients [104].

Percutaneous cryoablation has been widely used as a minimally invasive palliative treatment technique to treat various solid tumors. This method destroys the tumor tissue through direct freezing. According to previous studies, local freezing directly destroys the tumor cells, causes the release of TAAs in situ, and stimulates the antitumor immune response [105]. Traditional Chinese medicine for tumor treatment is guided by the “protective arena” theory, indicating that the immune mechanism around the tumor limits tumor growth. Therefore, when treating unresectable tumors, the surrounding tissue should be preserved [106]. Modern medicine supports this theory of traditional medicine. For example, in infiltrated-excluded TIMEs, CTLs only develop along the border of the tumor mass. TLSs are generally located at the invasive tumor margin, and their presence is often associated with a better prognosis [48]. In addition, tissue-resident memory CD8^+^ T cells are usually present in TLSs surrounding the tumors [107]. In one of our studies, percutaneous cryotherapy was used to partially destroy the cancerous tissue in the core of the tumor while minimizing damage to the surrounding tissues. This treatment protocol was used in combination with traditional Chinese medicine to treat 82 patients with advanced NSCLC. The average overall survival period was 18 months (13.12-22.88 months), which is better than that previously reported in patients receiving conventional treatments [108].

Many experimental studies have found that regional treatment of tumors disrupts the tumor cells, causes the release of TAAs, and changes the TIME, thus achieving antitumor effects [109, 110]. Although some clinical studies have shown that stereotactic radiation therapy, injection of α-GalCer-pulsed APC, and percutaneous cryotherapy exert a better therapeutic effect on NSCLC compared with conventional treatment methods, studies regarding the effects of these treatments on TIME are still limited.

## Concluding remarks and future perspectives

The occurrence of tumors indicates the failure of the immune surveillance function of the body and success of the tumor immune escape process. In addition to the direct elimination of tumor cells through surgery, radiotherapy, chemotherapy, and other methods, immunotherapy has become a breakthrough in cancer treatment. A particularly important example is ICIs. The study of TIME has enabled the direct observation of opposing immune processes, although only the tumor escape phase could be observed. Research on TIME has facilitated the discovery of new ways to inhibit tumor immune escape and help the body restore its immune surveillance functions. This article provides a comprehensive review of the clinical research on the interaction between the TIME and NSCLC therapies. The conclusions are summarized as follows: 1) Certain chemotherapy regimens, such as NAC, upregulate the PD-L1 expression in NSCLC cells, and combined ICI therapy is suitable for these patients. 2) NSCLC patients with high CD8^+^ TIL density in the tumor tissue prior to treatment respond better to chemotherapy and ICI treatment. ICI therapy increased the CD8^+^ T-cell infiltration rate in these patients. 3) Combination ICI therapy after EGFR-TKI treatment should be considered in patients with NSCLC carrying EGFR mutations. 4) NSCLC patients with different EGFR mutations or other mutations respond differently to treatment; thus, individualized treatment is vital. 5) Several studies have found that chemoradiotherapy increases the density of CD8^+^ lymphocytes, which is significantly associated with better prognosis. 6) Regional treatment of unresectable tumors, such as stereotactic radiotherapy and percutaneous cryotherapy, may help minimize damage to the tissues surrounding the tumor and promote the antitumor immune response. A limited number of clinical studies have shown that these approaches provide better results compared with conventional treatments; therefore, further research is needed to confirm this finding.

Based on the conclusions described above, future research on NSCLC treatment should consider the following four aspects. First, many clinical studies have indicated that combination therapy often achieves better therapeutic effects. Hence, future studies should evaluate the effects of a combination of treatment options, including surgery, radiotherapy, chemotherapy, targeted therapy, ICI therapy, and other immunotherapies. Second, with the rapid development of next-generation gene sequencing technology and information technology, the medical model is shifted from the traditional “one-size-fits-all” approach to a more tailored, precision medicine approach based on the specificity of genetic, environmental, and lifestyle factors in order to develop individualized treatment and prevention plans. Therefore, analyzing the personal health data of patients with lung cancer along with the genetic data of tumors, combined with information processing, will enable the development of personalized treatment plans in the future. Third, for older patients or individuals with advanced-stage disease, treatment methods should be developed based on treatment efficacy, minimal invasiveness, quick recovery, and high tolerability, such as the regional treatment of tumors. The purpose of research in this area is to prolong the patient’s survival and promote a high quality of life rather than simply removing the cancerous tissue. This treatment concept is called “green cancer therapy” [111]. Finally, the nutritional status of the patient is closely related to the TIME status and prognosis [112-114]. Therefore, the effect of immunonutrition on the TIME of NSCLC should be explored further in the future. In summary, studies on TIME and the progress made in immunotherapy indicate that restoring the immune surveillance function of the body is as important as removing the cancerous tissue itself.
